# Mediterranean Diet and Ultra-Processed Food Intake in Older Australian Adults—Associations with Frailty and Cardiometabolic Conditions

**DOI:** 10.3390/nu16172978

**Published:** 2024-09-03

**Authors:** Daniel Clayton-Chubb, Nicole V. Vaughan, Elena S. George, Andrew T. Chan, Stuart K. Roberts, Joanne Ryan, Aung Zaw Zaw Phyo, John J. McNeil, Lawrence J. Beilin, Cammie Tran, Yiqing Wang, Magdalena Sevilla-Gonzalez, Dong D. Wang, William W. Kemp, Ammar Majeed, Robyn L. Woods, Alice J. Owen, Jessica A. Fitzpatrick

**Affiliations:** 1 Department of Gastroenterology, Alfred Health, 99 Commercial Rd, Melbourne 3004, Australia; 2 School of Translational Medicine, Monash University, Melbourne 3004, Australia; 3 Department of Gastroenterology, Eastern Health, Box Hill 3128, Australia; 4 Department of Gastroenterology, St. Vincent’s Hospital Melbourne, Fitzroy 3065, Australia; 5 Department of Nutrition & Dietetics, Alfred Health, Melbourne 3004, Australia; 6 Institute for Physical Activity and Nutrition (IPAN), School of Exercise and Nutrition Sciences, Deakin University, Geelong 3220, Australia; 7 Clinical and Translational Epidemiology Unit, Massachusetts General Hospital, Boston, MA 02114, USA; 8 Division of Gastroenterology, Massachusetts General Hospital, Boston, MA 02114, USA; 9 Department of Medicine, Harvard Medical School, Boston, MA 02115, USA; 10 School of Public Health and Preventive Medicine, Monash University, Melbourne 3004, Australiaalice.owen@monash.edu (A.J.O.); 11 Medical School, Royal Perth Hospital, University of Western Australia, Perth 6000, Australia; 12 Programs in Metabolism and Medical & Population Genetics, The Broad Institute of MIT and Harvard, Cambridge, MA 02142, USA; 13 Channing Division of Network Medicine, Department of Medicine, Brigham and Women’s Hospital, Harvard Medical School, Boston, MA 02115, USA; 14 Department of Nutrition, Harvard T.H. Chan School of Public Health, Boston, MA 02115, USA

**Keywords:** epidemiology, dietary patterns, healthy ageing, nutrition, frailty

## Abstract

Dietary patterns contribute to overall health and diseases of ageing but are understudied in older adults. As such, we first aimed to develop dietary indices to quantify Mediterranean Diet Score (MDS) utilisation and Ultra-processed Food (UPF) intake in a well-characterised cohort of relatively healthy community-dwelling older Australian adults. Second, we aimed to understand the relationship between these scores and the association of these scores with prevalent cardiometabolic disease and frailty. Our major findings are that in this population of older adults, (a) pre-frailty and frailty are associated with reduced MDS and increased UPF intake; (b) adherence to MDS eating patterns does not preclude relatively high intake of UPF (and vice versa); and (c) high utilisation of an MDS eating pattern does not prevent an increased risk of frailty with higher UPF intakes. As such, the Mediterranean Diet pattern should be encouraged in older adults to potentially reduce the risk of frailty, while the impact of UPF intake should be further explored given the convenience these foods provide to a population whose access to unprocessed food may be limited due to socioeconomic, health, and lifestyle factors.

## 1. Introduction

Adults aged over 70 years are now more likely to live substantially longer compared to just two decades ago [1]. This, coupled with a reduction in fertility rates [2], has resulted in a higher distribution of older people commonly referred to as an ‘aging population’ [1]. Indeed, older persons represented only 5% of the global population in 1950—but this proportion is expected to increase to 16% by 2050 [1]. Despite this increase in life expectancy, disability burden has remained significant [3] and constant [1], and is associated with significant economic cost [4]. Data from the USA show that government health spending in older adults doubles between the ages of 70 and 90 [5], and Australian modelling data suggest that ageing-related increases in health expenditure from 2015 to 2035 may increase from AUD 166 billion to AUD 320 billion [6]. These increases will not be solely borne by higher-income countries—low- and middle-income countries are expected to have growing rates of multimorbidity and non-communicable disease with a concomitant increase in health expenditure [7]. This multimorbidity is a key driver not only of healthcare costs in older adults but also reduced quality of life [8,9,10,11]. Efforts to prevent this may assist in ameliorating the rapidly growing healthcare burden worldwide.

Many of these morbidities are non-communicable in nature and are related to changing dietary and lifestyle patterns. While dietary intake is widely recognised as a fundamental driver of health status [12], it is less well explored in older populations. Some data suggest that low vegetable and overall fibre intake are associated with worsened total disability [1] in people aged over 70, and conversely, that healthier dietary patterns are associated with improved quality of life [13]. Additionally, while some work has previously shown that dietary patterns are relatively stable in older adults [14], there are recognised barriers to achieving and maintaining ‘healthy eating’ [15], and understanding the potential benefits of promoting this—and, indeed, what constitutes ‘healthy eating’ in older adults—remains understudied.

In light of this focus on healthy eating in both middle-aged and older populations, there has been an increasing focus on ‘patterns’ of eating, rather than the historic approaches to studying single nutrients in isolation [16,17]. One such pattern is commonly termed the ‘Mediterranean Diet’ (MedDiet), a dietary pattern described by Ancel Keys in 1975 [18] and which has subsequently had an explosion of research showing associations with reduced all-cause mortality [19], cardiovascular disease [20,21], depression [22,23], cognitive impairment [22,24], and overall healthier ageing [25]. This dietary pattern is high in fat, rich in mono- and polyunsaturated fatty acids, polyphenols, fibre, and seafood, with only moderate amounts of other animal proteins. The diet has been evaluated both in geographically Mediterranean countries and countries beyond this region where the Mediterranean Diet is not habitual [26]. Different scores have been created to evaluate adherence to the Mediterranean Diet (MedDiet) in various regions. These scores are tailored to fit the available dietary data and account for specific cultural exclusions, inclusions, and the local food environment. Approaches to calculate scores vary but may include estimating fatty acid ratios, negatively scoring discretionary foods, and breaking down vegetable intake into different components such as green leafy vegetables and legumes [26]; however, it has not been explored extensively in older adults.

More recently, new ways to examine dietary intake have emerged. These include evaluating diets for ultra-processed foods (UPFs) defined via the NOVA classification [27,28]. UPF can be classified as packaged food that has undergone processing including the addition of food additives to improve cosmetic appearance, shelf-life, and palatability, such as sugar-sweetened beverages, confectionary, and canned goods [28]. Generally, UPF-rich diets are of a relatively poorer dietary quality overall [29], and recent studies have linked an increased intake of UPFs to an increased risk of all-cause mortality [30,31]. However, while some data suggest that UPFs account for up to 53% of total dietary energy intake in adults over 60 years of age in the USA [32], there are less data published on the associations between UPF intake and older adult morbidities. This is of particular importance given that while UPFs are often energy dense [33] and linked to overweight and obesity [33,34], the relative risk of poorer outcomes attributable to higher BMI is often ameliorated in older adults [35,36]. Therefore, it is possible that the associations between UPF intake and disease are attenuated in older adults due to the potential benefit in maintaining nutritional reserve (higher BMI) in this age group. Similarly, the hyper-palatability, extended shelf-life, and convenience inherent to many UPFs may have particular benefits for older adults with decreasing appetites and energy intake and who are at risk of malnutrition and frailty [37].

Furthermore, there are limited data on the ‘interaction’ between different dietary patterns. It is possible to have relatively strong adherence to a MedDiet while also consuming relatively high levels of UPFs, particularly if only using scores with ‘positive’ characteristics (e.g., high intake of vegetables, olive oil, and seafood lead to higher scores without reducing adherence for higher intakes of discretionary foods). There are also less well described data on what drives MedDiet and UPF intake differences in older adults, particularly in an Australian setting. 

As such, we aimed to (1) develop MedDiet and UPF scoring tools for use in a population of community-dwelling older Australian adults; (2) determine the relationship between the scores generated from these tools with prevalent cardiometabolic diseases and markers of frailty; (3) evaluate the constituent food types that drive higher scores in this cohort; and (4) evaluate for the relationship between these food and diet patterns to determine whether higher MedDiet intake is associated with lower UPF intake in older adults.

## 2. Methods

This is a secondary analysis of the ASPirin in Reducing Events in the Elderly (ASPREE) randomised trial [38] and the ASPREE Longitudinal Study of Older Persons (ALSOP) cohort study [39]. The baseline characteristics of these studies, the trial protocol, and the primary outcomes have been reported previously [38,39,40,41,42]. In brief, 16,703 Australian participants were recruited via primary care for enrolment in a randomised trial of 100 mg of aspirin vs. placebo for reducing the risk of disability-free survival, physical disability, dementia, mortality, and cardiovascular disease. Key inclusion criteria in Australia included being aged 70 or more years, being willing and able to provide informed consent, and being willing to accept the study requirements. Key exclusion criteria at baseline included the following: self- or physician-reported dementia, a Modified Mini-Mental State examination (3MS) [43] less than 78, established or previous cerebrovascular or cardiovascular disease, a lack of functional independence in 1 or more of 6 basic activities of daily living [38,44], and/or a serious illness likely to cause death within 5 years. Subsequently, during the trial, the ALSOP cohort study [39] was initiated to further examine lifestyle factors in these older Australian adults with three waves of questionnaire data collection. The third wave data are not yet available. The second wave (approximately 3 years after the commencement of ASPREE) included a 54-item food frequency questionnaire and was the basis for this study. We excluded participants who were no longer living in the community (either alone, or with friends/family/spouse) at the time of the ALSOP questionnaire, as a change in living situation may have led to a change in dietary pattern and food choices may be dictated by providers in higher care settings. All participants were followed up with both annual in-person visits and medical record reviews, and between-visit telephone calls were also conducted. All participants provided written and informed consent, and the ASPREE trial and the ALSOP were approved by local ethics committees. The ASPREE trial is registered on ClinicalTrials.gov (NCT01038583) and the International Standard Randomized Controlled Trial Number Registry (ISRCTN83772183).

### 2.1. Baseline and Longitudinal Participant Assessment

At both baseline and during the ASPREE study follow-up, in-person interviews and assessments were conducted by trained study staff to gather information on medical and social history, lifestyle, cognitive and functional assessments, anthropometric measurements, and laboratory markers. These included evaluation of cognitive function via the 3MS [43], the Center for Epidemiologic Studies Depression Scale (CES-D-10) [45,46], and annual fasting glucose and lipid parameters performed at local pathology centres. Anthropometric and functional measures included abdominal circumference, weight, height, BMI, grip strength (using a spring-type or hydraulic hand-held dynamometer [Jamar, Chicago, IL, USA; Lafayette Instruments, Lafayette, IN, USA] three times and averaged for the dominant hand) while seated, and gait speed (habitual walking pace over 3 metres measured twice and averaged).

### 2.2. Dietary Score Questionnaire Development

The 54-item food-frequency questionnaire (FFQ) (Supplementary Table S1) from the second wave of the ALSOP [39] was used to generate the two dietary scores. The FFQ only recorded foods via frequency of consumption with no portion size of food recorded. Therefore, energy (kilojoule) and macro- or micronutrient intake were not able to be calculated. Firstly, for the UPF score (ASPREE-UPF), the full list was independently evaluated by DCC and JAF with each item classified as a UPF or not using the NOVA classification system (NOVA 4) as previously described both internationally [28,47] and in Australia [48]. Discordance was resolved through further review of the relevant UPF literature and input from a third author (PPS). Twenty-five foods/beverages were thus classified as UPFs where each was given 1 point for maximal intake based on the FFQ and lesser points for lower frequency. The total ASPREE-FFQ is the sum of each score attributable to each of the 25 UPF items (Supplementary Table S2). Valid FFQs were defined as questionnaires that had at least 20 of the 25 UPF items for the ASPREE-UPF, otherwise the participant was excluded. Missing values were otherwise given the overall population median score for that line. The maximum ASPREE-UPF score (i.e., highest level of UPF exposure) is 25, and the minimum is 0. Associations were made with the ASPREE-UPF score as a continuous variable and when stratified into quartiles.

For the MedDiet score (ASPREE-MDS), the full ASPREE 54-item FFQ was independently evaluated by DCC and JAF and classified as contributing to or detracting from MedDiet principles [18,49]. In keeping with some of the scores previously used, an ‘optimal’ ASPREE-MDS was determined as only being possible when excluding significant discretionary food intake. The included line items were determined by consensus and discussion, and author ESG provided subsequent review and additional research to inform this consensus methodology. Subsequently, line-item scores were decided based on a review of the MedDiet literature with a focus on vegetable/fruit/grain diversity, olive oil as the predominant vegetable oil, additional points for oily fish intake, and adequate nuts and legumes (Supplementary Table S3). Valid FFQs were defined as questionnaires that had at least 30 of the 38 included line items for the ASPREE-MDS completed, otherwise the participant was excluded. Missing values were otherwise replaced with the overall population median score for that line. The maximum MedDiet adherence score possible with the ASPREE-MDS is 18, with a minimum score possible of 0. Analysis was performed with the ASPREE-MDS both as a continuous score and stratified into quartiles.

### 2.3. Morbidity Definitions

All the variables used were taken from the study visits most proximate to the time the second wave ALSOP questionnaire was returned. If the data for that variable were missing, the response from the year prior was recorded in its place. If a data point was missing from both of those time points it was excluded for that participant. Dyslipidaemia was defined as a total cholesterol ≥ 200 mg/dL and/or LDL cholesterol ≥ 130 mg/dL and/or triglycerides ≥ 150 mg/dL and/or HDL cholesterol < 40 mg/dL (males) or <50 mg/dL (females) and/or prescription of lipid lowering therapy [50]. As previously described in the ASPREE population, type 2 diabetes mellitus (T2DM) was defined as a glucose ≥ 126 mg/dL and/or the prescription of a glucose lowering medication [51,52], hypertension was defined as a systolic blood pressure ≥ 140 mmHg and/or a diastolic blood pressure ≥ 90 mmHg and/or prescription of an antihypertensive(s) [51], and chronic kidney disease (CKD) was defined as an eGFR < 60 mL/min/1.73 m^2^ and/or a urine albumin/creatinine ratio ≥ 30 mg/g [51]. Low gait speed was defined as ≤0.8 m/s [52,53]. Low grip strength was defined as <16 kg (for females) and <27 kg (for males) [52,53]. Additionally, the ASPREE Deficit-Accumulation Frailty Index was calculated and scored as previously described [54]. The index was constructed based on the method of Rockwood et al. [55]. It consists of 67 items covering 11 comorbidities, 13 disease indicators including anaemia and central adiposity, 26 functional deficits interfering with completing activities of daily living, 11 mental and psychosocial deficits, and 6 cognitive function and physical performance (grip strength and gait speed) items [54]. The frailty index was calculated as the average number of deficits across all items, with participants classified as non-frail, pre-frail, or frail. This frailty index has been previously shown in the ASPREE population to stratify groups at risk of reduced disability-free survival [54]. BMI cut-offs were stratified as underweight < 23 kg/m^2^, normal weight 23–28 kg/m^2^, overweight 28–33 kg/m^2^, or obese ≥ 33 kg/m^2^ due to different optimal BMIs for older adults than younger adults [35]. Central adiposity was defined as ≥88 cm (female) and ≥102 cm (male) [56]. A CES-D-10 score of ≥8 signified depressive symptoms [57].

### 2.4. Statistics

Descriptive statistics were presented as mean and standard deviation or median and interquartile range, depending on distribution of data. Baseline demographic data were evaluated using a Chi-square test, Student’s *t* test, one-way ANOVA, Mann–Whitney U, or Kruskall–Wallis tests as appropriate. Initially, demographic differences and differences in the ASPREE-MDS and ASPREE-UPF were stratified by sex due to previous data suggesting differences in adherence to healthy eating patterns in males and females [58,59,60]. Subsequently, relationships of these scores in quartiles with markers of frailty and cardiometabolic comorbidities (including T2DM, CKD, hypertension, and dyslipidaemia) were explored. Finally, logistic regression adjusted for age, sex, educational attainment, BMI, living situation, smoking status, and alcohol drinking status was used to evaluate the relationship between these scores and morbidities of interest. Multinomial logistic regression was similarly used (similarly adjusted) to explore the relationship between the ASPREE-MDS and ASPREE-UPF with pre-frailty and frailty defined using the Deficit-Accumulation Frailty Index. A *p* < 0.05 was considered significant. Statistical analyses were performed using Stata software v17.0 (StataCorp LLC, College Station, TX, USA).

## 3. Results

### 3.1. Study Population

**Figure 1:**
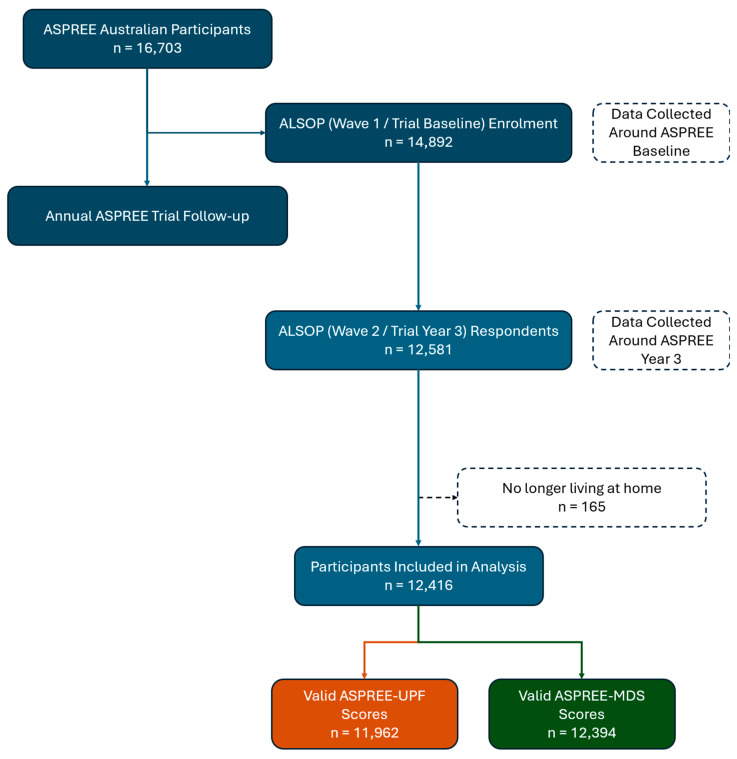
Patient flow chart.

Out of 12,581 respondents to the second wave of the ALSOP questionnaire, 165 were no longer independently living in the community (either alone or with friends/family/spouse) and so were excluded, leaving 12,416 participants for score development and evaluation (Figure 1). These participants had a median age of 76.9 (IQR 74.6–80.3) years, 6751 (54.4%) were females, and the mean (SD) BMI was 27.6 (±4.6) kg/m^2^. The females were slightly older (77.0 [IQR 74.7–80.5] vs. 76.7 [IQR 74.5–80.1] years, *p* < 0.001), less likely to be current drinkers (67.1% vs. 81.5%, *p* < 0.001), more likely to be home alone (44.5% vs. 19.5%, *p* < 0.001), and less likely to have completed 13 or more years of education (37.6% vs. 44.5%, *p* < 0.001). The BMI distribution for women was wider than for men, with both higher rates of BMI < 23 (17.8% vs. 9.5%) kg/m^2^ and BMI > 33 (14.3% vs. 8.8%) kg/m^2^, higher rates of central adiposity (63.2% vs. 46.6%, *p* < 0.001), and higher rates of dyslipidaemia (88.1% vs. 75.8%, *p* < 0.001) but lower rates of T2DM (8.4% vs. 12.7%, *p* < 0.001). There were more pre-frail and frail female than male participants (56.3% vs. 40.4%, *p* < 0.001) (Table 1).

### 3.2. ASPREE-MDS

**Figure 2:**
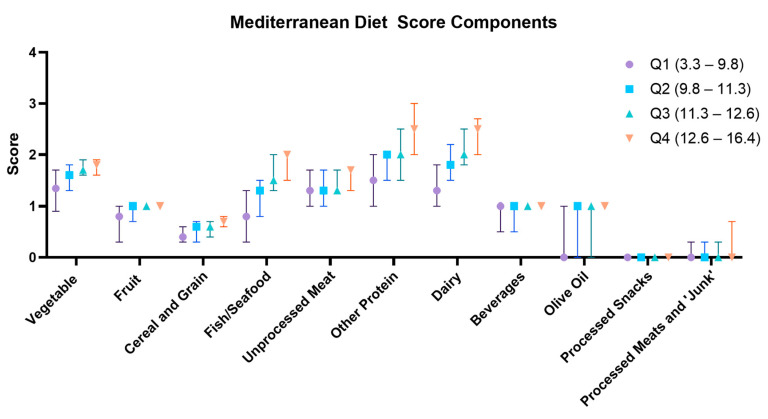
Contributors to ASPREE-MDS by quartile.

The ASPREE-MDS was generated for 12,394 participants with valid ALSOP FFQ responses who had a median score of 11.3 (IQR 9.8–12.6) out of a maximum possible of 18. The ASPREE-MDS was generally higher in females than males (11.6 [IQR 10.3–12.9] vs. 10.9 [IQR 9.4–12.2], *p* < 0.001) (Table 1), and each food component significantly contributed to the overall ASPREE-MDS (Table 2). In particular, comparing the 4th to the 1st ASPREE-MDS quartile, vegetable (median 1.8 [IQR 1.6–1.9] vs. 1.3 [IQR 0.8–1.7], *p* < 0.001), fish/seafood (median 2.0 [IQR 1.5–2.0] vs. 0.8 [IQR 0.3–1.3], *p* < 0.001), non-meat non-seafood protein (median 2.5 [IQR 2.0–3.0] vs. 1.5 [IQR 1.0–2.0], *p* < 0.001), and dairy (median 2.5 [IQR 2.0–2.8] vs. 1.3 [1.0–1.8], *p* < 0.001) intake each meaningfully individually contributed to the overall ASPREE-MDS. Processed snack intake (Q4 median 0.0 [IQR 0.0–0.0] vs. Q1 median 0.0 [IQR 0.0–0.0]) or processed meat and discretionary food intake (Q4 median 0.0 [IQR 0.0–0.7] vs. Q1 median 0.0 [IQR 0.0–0.0]) did not alter the overall scores in a numerically meaningful way (Table 2, Figure 2). While the sex-stratified score distributions were generally similar, the relatively small sex difference was predominantly attributable to females having slightly higher vegetable scores, higher dairy scores, and higher seafood scores (Table 2).

Those with higher ASPREE-MDS —representing greater adherence to a Mediterranean pattern of eating—were more likely to be female (Q4 65.8% vs. Q1 41.7%, *p* < 0.001), have completed at least 13 years of formal education (Q4 49.7% vs. Q1 31.3%, *p* < 0.001), score higher on the 3MS (Q4 95.0 ± 4.3 vs. Q1 93.3 ± 5.2, *p* < 0.001), and be less likely to have a CESD-10 score consistent with possible depression (Q4 14.8% vs. Q4 18.4%, *p* = 0.003) (Table 3). There was no significant difference in those living alone vs. living with others across the ASPREE-MDS quartiles (*p* = 0.057) (Table 3). Interestingly, while there was a statistically significant difference in ASPREE-UPF scores across the ASPREE-MDS quartiles, these differences were numerically slight (Q4 median 6.1 [IQR 5.0—7.3] vs. Q1 5.9 [IQR 4.8—7.1], *p* < 0.001).

### 3.3. ASPREE-UPF

**Figure 3:**
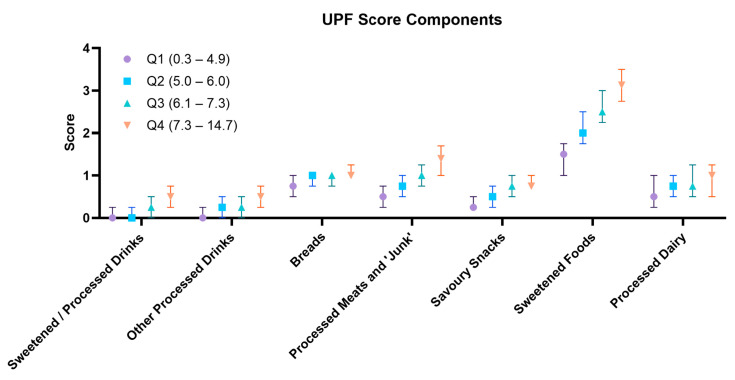
Contributors to ASPREE-UPF by quartile.

The ASPREE-UPF was able to be calculated for 11,962 ALSOP FFQ participants and had a median score of 6.0 (IQR 4.9–7.3) with a maximum possible score of 25. The ASPREE-UPF scores were lower in females than males (5.8 [4.8–7.0] vs. 6.5 [5.3–7.7], *p* < 0.001) (Table 1). Each UPF intake sub-type contributed to the overall UPF score (Table 4, Figure 3). Comparing the 4th to the 1st ASPREE-UPF quartile, the numerical differences between processed meats and junk foods (median 1.4 [IQR 1.0–1.7] vs. median 0.5 [IQR 0.25–0.75], *p* < 0.001) and sweetened foods (median 3.1 [IQR 2.8–3.5] vs. 1.5 [IQR 1.0–1.8], *p* < 0.001) were particularly large. Comparatively, however, the ‘bread’ category had a numerically smaller difference across the quartiles (Q4 median 1.0 [IQR 1.0–1.3] vs. Q1 median 0.8 [IQR 0.5–1.0], *p* < 0.001) (Table 4, Figure 3). When considering the sex difference in the ASPREE-UPF score, males scored higher on sweetened/processed beverages and consumed more bread, processed meats and related foods, savoury snacks, and sweetened foods (Table 4).

**Figure 4:**
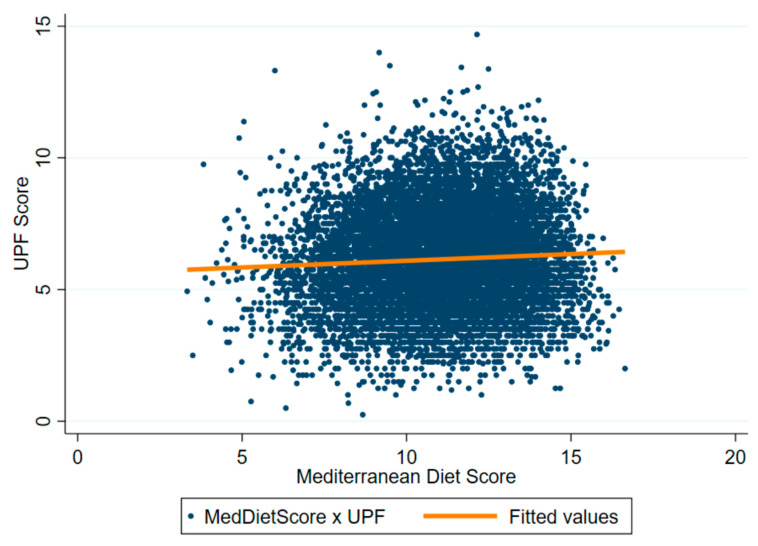
Relationship between ASPREE-MDS and ASPREE-UPF.

Those with the highest ASPREE-UPF scores compared with the lowest (Q4 vs. Q1) were less likely to be female (41.1% vs. 64.5%, *p* < 0.001), less likely to live alone (26.9% vs. 39.2%, *p* < 0.001), and were more likely to have a CESD-10 score potentially consistent with depression (19.0% vs. 14.9%, *p* < 0.001) (Table 5). The ASPREE-MDS distribution was similar across the ASPREE-UPF quartiles (Table 5). There was only a very weak positive relationship between ASPREE-MDS and ASPREE-UPF (r^2^ = 0.057, 95% CI 0.039—0.075) (Figure 4), suggesting that increasing Mediterranean Diet adherence does not necessarily mean markedly less UPF consumption.

### 3.4. Dietary Patterns with Cardiometabolic Diseases and Frailty Markers

On univariate analysis while stratifying by quartiles and comparing Q4 to Q1, the ASPREE-MDS was associated with fewer BMI > 33 kg/m^2^ (10.2% vs. 13.0% *p* < 0.001), less central adiposity (52.2% vs. 57.4%, *p* < 0.001), less T2DM (9.1% vs. 11.9%, *p* < 0.001), less hypertension (70.0% vs. 76.8%, *p* < 0.001), and less CKD (25.0% vs. 33.6%, *p* < 0.001) but marginally higher dyslipidaemia (83.2% vs. 80.5%, *p* = 0.005) (Table 3). There was also less frailty (9.4% vs. 13.6%) and more non-frailty (56.2% vs. 46.7%) when comparing Q4 to Q1.

In contrast, when comparing Q4 to Q1 for the ASPREE-UPF score, higher UPF intake was associated with fewer BMI < 23 kg/m^2^ (12.8% vs. 16.2%, *p* < 0.001), similar amounts of central adiposity (53.6% vs. 56.1%, *p* = 0.013), and no difference in the proportion with T2DM (10.0% vs. 9.8%), hypertension (72.9% vs. 74.2%), or CKD (30.0% vs. 28.3%) (Table 5). There was no significant difference in frailty between quartiles (*p* = 0.183).

When utilising logistic regression adjusted for age, sex, BMI, educational attainment, living situation, and current smoking and alcohol drinking status, higher ASPREE-MDS was associated with reduced odds of CKD (aOR 0.94 [95% CI 0.92–0.96]) and hypertension (aOR 0.96 [95% CI 0.94–0.98]). Furthermore, using multinomial logistic regression, higher ASPREE-MDS was associated with a decreased risk of both pre-frailty (aRR 0.93 [95% CI 0.91–0.95]) and frailty (aRR 0.88 [95% CI 0.86–0.91]) (Table 6). Similarly, a higher ASPREE-UPF score was associated with lower odds of hypertension (aOR 0.97 [95% CI 0.94–0.99]) (Table 6). There was a weak association between higher ASPREE-UPF score and frailty on unadjusted analysis (RR 1.04 [95% CI 1.00–1.07], *p* = 0.023), which strengthened for the fully adjusted analysis (pre-frailty aRR 1.04 [95% CI 1.01–1.06] and a frailty aRR of 1.10 [95% CI 1.06–1.14]). In a multinomial logistic regression model including both scores concurrently, the pre-frailty and frailty results were similar (Table 6). In this fully adjusted analysis, there was no significant association between either ASPREE-MDS or ASPREE-UPF with prevalent T2DM or dyslipidaemia (*p* > 0.05) (Table 6).

## 4. Discussion

There is widespread scientific consensus and a significant body of evidence to support the use of the MedDiet as a protective dietary pattern and a possible contributor to healthier ageing [25]. However, adherence to these dietary patterns in older adults is understudied, as is their association with major age-related morbidities such as cardiometabolic diseases and markers of frailty. Similarly, there is a paucity of research looking at the relationship between different diet patterns and compositions of food intake. Understanding these relationships may have public health implications such as holistic dietary recommendations that address variable styles of eating. To examine these factors, we developed dietary scores from FFQs in the Australian ASPREE/ALSOP cohort, to evaluate both adherence to a MedDiet and concurrent rate of UPF intake. Our primary findings are firstly that, in this population of community-dwelling older Australian adults, a Mediterranean dietary pattern is contributed to by a variety of different healthy dietary components; secondly, while a Mediterranean dietary pattern adherence differs by sex, no single food category drives this difference; third, UPF intake is relatively common in older people and is driven by “discretionary food”; fourth, there is a weak relationship between these two dietary scores in this study (where high UPF intake does not preclude adherence to a Mediterranean style of eating and vice versa); and finally, these dietary patterns have opposing impacts on frailty. High Mediterranean dietary score adherence is associated with reduced odds of hypertension, pre-frailty and frailty, and CKD, and high UPF scores are associated with frailty but a lower risk of hypertension.

## 5. Mediterranean Diet in Older Adults

The MedDiet was initially developed and studied in regions surrounding the Mediterranean Sea. In more recent years, its beneficial effects have been demonstrated in studies in many other Western countries, including the United States, Australia, and the United Kingdom [61]. Multiple previous studies have focused on cardiometabolic benefits [62], and these have been explored in a variety of meta-analyses. A meta-analysis of 19 randomised controlled trials in adults across the age spectrum (median age 53.0 [range 25.0–70.9] years) described an association between MedDiet adherence and reduced risk of developing hypertension (OR 0.87 [95% CI 0.78–0.98]) [63]. Similarly, prior studies have shown that MedDiet adherence is associated with both reduced incident CKD and reduced CKD progression [64,65], though the majority of the work in these areas has been in middle-aged adults. Despite the different age groups studied, these data concord with our results in our markedly older population, where reduced odds of both hypertension (aOR 0.96 [95% CI. 94–0.98]) and CKD (aOR 0.94 [95% CI 0.92–0.96]) were seen with higher ASPREE-MDS.

More recent work has also evaluated the role of the MedDiet in physical health and frailty in older adults. Results from our study are consistent with the findings of two recent meta-analyses of both cohort and observational studies, where a reduction in risk of both prevalent [66] and incident [67] frailty in older adults (greater than 60 years of age) was found to be associated with increasing adherence to the MedDiet. In the Australian setting, the relationship between dietary patterns and frailty was also explored in a prospective cohort study, where it was found that a diet rich in vegetables, legumes, and seafood (core MedDiet components) was associated with reduced prevalence of frailty in older community-dwelling Australian men [68]. These data are in keeping with our findings, where higher adherence to the MedDiet using the ASPREE-MDS was associated with a reduced risk of both pre-frailty (aRR 0.93 [95% CI 0.91–0.95]) and frailty (aRR 0.88 [95% CI 0.86–0.91]), underscoring the potential benefit of the MedDiet in supporting healthy ageing.

There are multiple proposed explanations exploring the effects of the MedDiet on cardiometabolic markers and overall healthy ageing. In the older population, microbiome modulation has been proposed as one possible driver. One study has identified that increased adherence to the MedDiet was coupled with an increase in the abundance of specific microbiotal taxa known to be positively associated with several markers of lesser frailty, lower systemic inflammation, and improved cognitive function in older adults [69]. Another plausible explanation includes specific dietary components. Olive oil, for example, may act at the genomic level by modulating systemic inflammation and oxidative stress, thus effecting epigenetic change [70]. Additionally, as highlighted in our study, an integral component of the MedDiet is the contribution of non-meat and non-seafood proteins, such as legumes. Legumes, in addition to being an important source of fibre and complex carbohydrates, contain protein and are low in saturated fats [71]. Reduction in dietary saturated fats and replacement with food sources rich in unsaturated fats or carbohydrates is a well-established dietary strategy shown to reduce risk of cardiovascular events by reducing the impact of saturated fat as a driver for increased serum cholesterol [72]. Given this, some of the benefits of the MedDiet may also be about what is not included, rather than what is. In our cohort, however, there was no meaningful contribution to the ASPREE-MDS through avoidance of discretionary foods (which are often high in saturated fat and added sugar and lower in antioxidants and fibre). As such, in this population of older adults, the positive MedDiet associations seem to be—at least in part—contributed to by the overall variety of polyphenols, fibre, mono- and poly-unsaturated fats, and lean proteins seen with higher ASPREE-MDS scores.

## 6. UPF Intake in Older Adults

UPFs are food products that include ingredients that have undergone processing techniques including cosmetic additives (e.g., colours, flavours, emulsifiers, thickeners, and artificial sweeteners) or oils, fats, sugar and protein derivatives (e.g., homogenised fats, protein isolates, maltodextrin, and high-fructose corn syrup) as classified by the NOVA system [27]. Compared to less processed foods, UPFs are generally more energy dense [47], contain higher levels of saturated and trans-fats, refined sugars, and salt [73] and are lower in dietary fibre [74]. The current literature links UPF consumption with poorer diet quality [29,74], yet UPFs may account for up to 53% of total dietary energy intake in older American adults [32]. In Australian older adults, estimates of UPF intake as a proportion of total dietary intake is lower yet remains substantial at 36% [75]. A recent umbrella review and meta-analysis found that high UPF consumption was associated with an increased risk of developing multiple chronic health and mental health conditions [76] including dementia [77], depression [78], diabetes [79], cardiovascular diseases [80], and all-cause mortality [76]. However, the majority of these studies included or were performed exclusively on middle-aged adults, with less of the literature evaluating the impact of UPFs on outcomes in older adults.

In a cross-sectional study conducted from a National Survey in people over 60 years old living in the United States, greater UPF contribution to total dietary energy intake was found to be positively associated with frailty risk in underweight, normal-weight, and overweight people [81]. Another study conducted in Italy found that eating more unprocessed or minimally processed foods was inversely associated with the presence of frailty in those aged 65 years or older [82]. This is also reflected in our study, where higher UPF intake as assessed via the ASPREE-UPF score—when adjusting for multiple covariates—was associated with an increased risk of pre-frailty (aRR 1.04 [95% CI 1.01–1.06]) and frailty (aRR 1.10 [95% CI 1.06–1.14]).

Several studies have demonstrated a link between increased UPF intake and risk of hypertension. However, studies exploring the association between UPFs and hypertension in older adults remain relatively limited; in a recent systematic review linking UPFs and hypertension, only three studies included older people [83]. Several key biological pathways have been proposed to explain this link, including alteration of serum lipid concentrations, modified gut microbiota and host–microbiota interactions, obesity, inflammation, and insulin resistance [84].

Interestingly, after performing a logistic regression and adjusting for age, sex, BMI, educational attainment, living situation, and current smoking and drinking status, our study demonstrated that higher ASPREE-MDS and ASPREE-UPF scores were both associated with reduced rates of hypertension. This link between higher UPF consumption and lower rates of hypertension has not been previously described in the literature. A comparison of dietary patterns in older Australians suggest poor dietary patterns are often accompanied by lower education attainment and other negative lifestyle behaviours such as smoking and low levels of physical activity [85]. This unexpected outcome may be partly explained by the adjustment for confounding risk factors, particularly excess body weight, which is inextricably linked to both high dietary intakes of UPF [75,86,87] and hypertension [88]. It is also possible that in this population, a higher ASPREE-UPF score is contributed to by homemade products such as cakes or biscuits, as the ASPREE FFQ does not delineate where these foods are made. If they were made at home, they would not be classified as UPF—this is a limitation of the FFQ design. Some homemade baked goods may vary significantly in their ingredients compared to UPF baked goods, as they would lack food additives and modified starches, and may contain ingredients higher in fibre such as oats, fruits, or vegetables and olive oil instead of butter or hydrogenated oils with associated potential health benefits [89,90]. Additionally, it is possible that the line of causality is reversed in this population of older adults who were sufficiently interested in their health to volunteer and be eligible for a primary prevention clinical trial—those with pre-existing hypertension may have modified their diets to consume less UPFs.

Another interesting finding from our study was that high UPF intake did not preclude high adherence to a MedDiet. This is contrary to the existing literature on younger adults [91], though the relationship between these two indices has not been previously explored in older adults. Furthermore, the data on higher adherence to a MedDiet pattern and inclusion of UPFs are mixed, with other work suggesting that these intakes are not mutually exclusive. One study involving university students found that consumption of ultra-processed plant-based meat alternatives was positively associated with MedDiet adherence [92], and while another has shown decreased MedDiet adherence with increased UPF intake [93], frequency of UPF intake has not been as well explored. In our study, we have shown that an increased frequency of UPF intake is only very weakly related to MedDiet adherence—it is possible that other results to the contrary are due to UPF intake being based on the total energy percentage attributable to UPFs (which we were unable to perform with our FFQs). It is also possible that, in our older population (median age of 76.8 years), the total energy consumption attributable to UPFs is relatively lower compared to younger adults, perhaps due to lower overall appetite and energy consumption in a proportion of the cohort [37]. Irrespectively, our results indicate that increased UPF intake frequency and increased MedDiet adherence can co-exist, and both have a significant independent association with pre-frailty and frailty.

## 7. Strengths and Weaknesses

Our study has numerous strengths, including its size and unique composition of older people, its rigorous and protocol-driven prospective data capture from inception through to follow-up, and its robust and broad dietary, anthropometric, and lifestyle questionnaires. However, some limitations should be discussed. Firstly, there were no data on quantities of food eaten—just frequencies. This both limited the opportunities to apply previously generated epidemiologic scores from other studies as well as limited our understanding of the actual quantities of important dietary components (e.g., olive oil and nuts) which would have supported interpretation of these scores and comparison to other similar scores. Secondly, the FFQ itself was based on self-report—while the prompt was to consider the previous 12 months of eating, there are known issues around dietary recall which may limit individual participant reporting accuracy. Third, this cohort may represent healthy volunteer bias, potentially skewing data away from population norms of UPF intake or MedDiet adherence; similarly, there may be unmeasured confounders impacting dietary intake. Fourth, there was no capacity to directly capture biochemical micronutrient data. Finally, this population was predominantly self-described Australian Caucasian, limiting its generalisability to other geographies and ethnicities. Despite these limitations, our scores showed similar demographic associations to those previously described in the literature; a high ASPREE-MDS was more common in females, more common in the highly educated cohort, and associated with lower levels of obesity. Similarly, a high ASPREE-UPF (sometimes consistent with a less-healthy diet) was less likely in females; in combination, these findings provide some reassurance that the score is functioning as intended.

## 8. Conclusions

In this sub-study of initially relatively healthy community-dwelling older adults living at home, we have shown that the generation of Mediterranean Diet adherence and UPF intake scores is feasible; that higher scores using the ASPREE-MDS are attributable to a diversity of food groups (including oily fish/seafood, vegetable intake, and non-meat non-seafood protein intake); and that in this population, there is a very weak relationship between Mediterranean Diet adherence and UPF intake. Furthermore, we have shown that even when adjusting for sociodemographic and lifestyle factors, ASPREE-MDS is associated with reduced rates of prevalent hypertension, CKD, and frailty. Similarly, higher UPF intake is associated with frailty. These data have important implications for counselling and public health messaging. Increasing Mediterranean Diet adherence is an actionable public health message and aligns with many principles of healthy eating. The causal relationship between UPF and frailty should be further explored given the convenience these foods provide to a population whose access to unprocessed food may be limited due to socioeconomic factors.

## Figures and Tables

**Table 1:** Participant demography.

	Females (*n* = 6751)	Males (*n* = 5665)	*p*
**Age (Median [IQR]), years**	77.0 (74.7–80.5)	76.7 (74.5–80.1)	<0.001 ^a^
**Caucasian Ethnicity, n (%)**	6680 (98.9%)	5584 (98.6%)	0.056 ^b^
**Education Completion**			<0.001 ^b^
≤11 years of education (n, %)	3457 (51.2%)	2554 (45.1%)	
12 years of education (n, %)	755 (11.2%)	588 (10.4%)	
≥13 years of education (n, %)	2538 (37.6%)	2523 (44.5%)	
**Lifestyle Factors**			
Currently Drinking Alcohol (n, %)	4528 (67.1%)	4615 (81.5%)	<0.001 ^b^
Currently Smoking (n, %)	151 (2.2%)	144 (2.5%)	0.265 ^b^
**Living Situation**			<0.001 ^b^
Home alone (n, %)	3001 (44.5%)	1103 (19.5%)	
Home with spouse/friends/family (n, %)	3750 (55.5%)	4562 (80.5%)	
**BMI** (mean ± SD), kg/m^2^	27.6 ± 5.1	27.6 ± 3.8	0.608 ^c^ <0.001 ^b^
**BMI Categories** (n, %)			
BMI < 23 kg/m^2^	1200 (17.8%)	539 (9.5%)	
BMI 23–28 kg/m^2^	2706 (40.2%)	2813 (49.7%)	
BMI 28–33 kg/m^2^	1781 (27.8%)	1809 (32.0%)	
BMI > 33 kg/m^2^	962 (14.3%)	500 (8.8%)	
**Abdominal circumference** (mean ± SD), cm	92.3 ± 12.7	101.4 ± 10.8	<0.001 ^c^
Central adiposity (abnormal abdominal circumference) (n, %)	4262 (63.2%)	2639 (46.6%)	<0.001 ^b^
**Laboratory Parameters**			
Glucose (mean ± SD), mg/dL	97.1 ± 16.4	102.0 ± 19.8	<0.001 ^c^
Total Cholesterol (mean ± SD), mg/dL	206.4 ± 38.0	187.2 ± 36.3	<0.001 ^c^
HDL Cholesterol (mean ± SD), mg/dL	67.6 ± 17.9	54.5 ± 15.0	<0.001 ^c^
LDL Cholesterol (mean ± SD), mg/dL	115.2 ± 35.0	109.5 ± 32.9	<0.001 ^c^
Triglycerides (mean ± SD), mg/dL	117.8 ± 53.0	116.3 ± 58.4	0.146 ^c^
eGFR (mean ± SD), mL/min/1.73 m^2^	70.4 ±14.1	70.5 ± 14.1	0.883 ^c^
**Cardiometabolic Conditions**			
T2DM, n (%)	564 (8.4%)	720 (12.7%)	<0.001 ^b^
Hypertension, n (%)	4929 (73.0%)	4215 (74.4%)	0.079 ^b^
Chronic Kidney Disease, n (%)	1961 (29.0%)	1655 (29.2%)	0.838 ^b^
Dyslipidaemia, n (%)	5946 (88.1%)	4296 (75.8%)	<0.001 ^b^
**Physical Function**			
Deficit-Accumulation Frailty Index			<0.001 ^b^
Not Frail	2934 (43.7%)	3364 (59.6%)	
Pre-Frail	2769 (41.2%)	1885 (33.4%)	
Frail	1015 (15.1%)	396 (7.0%)	
Gait Speed (mean ± SD), m/s	1.09 ± 0.3	1.0 ± 0.2	<0.001 ^c^
Low Gait Speed, n (%)	598 (8.9%)	725 (12.8%)	<0.001 ^b^
Grip Strength (mean ± SD), kg	20.1 ± 5.2	33.6 ± 8.0	<0.001 ^c^
Low Grip Strength, n (%)	1384 (20.5%)	1091 (19.3%)	0.078 ^b^
**Neurocognitive Health**			
3MS Score (mean ± SD)	94.9 ± 4.5	93.4 ± 5.1	<0.001 ^c^
CESD score ≥ 8, n (%)	1280 (19.6%)	745 (13.6%)	<0.001 ^b^
**Dietary Scores**			
ASPREE-MDS (Median [IQR])	11.6 (10.3–12.9)	10.9 (9.4–12.2)	<0.001 ^a^
ASPREE-UPF (Median [IQR])	5.8 (4.8–7.0)	6.5 (5.3–7.7)	<0.001 ^a^

BMI: body mass index, T2DM: Type 2 diabetes mellitus, 3MS: Modified Mini Mental State Examination Score, CESD: Center for Epidemiological Studies Depression Scale, MDS: Mediterranean diet score, UPF: Ultra-processed food. Data analysed via ^a^ Mann–Whitney U test, ^b^ Chi-square test, ^c^ Student’s *t*-test.

**Table 2:** ASPREE-MDS dietary components.

ASPREE-MDS Dietary Components	Q1 (3.3–9.8) (n = 3102)	Q2 (9.8–11.3) (n = 3096)	Q3 (11.3–12.6) (n = 3110)	Q4 (12.6–16.4) (n = 3086)	*p* ^a^	Female (Median: 11.6) (n = 6736)	Male (Medan: 10.9) (n = 5492)	*p* ^b^
Vegetable Intake Score (Includes green vegetables and other vegetables)	1.3 (0.9–1.7)	1.6 (1.3–1.8)	1.7 (1.6–1.9)	1.8 (1.6–1.9)	<0.001	Median: 1.7 Q1: 0.0–1.3 Q4: 1.9–2.0	Median 1.6 Q1: 0.0–1.3 Q4: 1.8–2.0	<0.001
Fruit Intake Score (Includes fresh and canned/tinned Fruits)	0.8 (0.3–1.0)	1.0 (0.7–1.0)	1.0 (1.0–1.0)	1.0 (1.0–1.0)	<0.001	Median: 1.0 Q1: 0.0–1.0 Q4: 1.0–1.0	Median: 1.0 Q1: 0.0–0.7 Q4: 1.0–1.0	<0.001
Cereal and Grain Intake Score (Includes brown bread, pasta, rice, and cereal)	0.4 (0.3–0.6)	0.6 (0.3–0.7)	0.6 (0.4–0.7)	0.7 (0.6–0.8)	<0.001	Median: 0.6 Q1: 0.0–0.3 Q4: 0.7–1.0	Median: 0.6 Q1: 0.0–0.3 Q4: 0.7–1.0	<0.001
Fish/Seafood Intake Score (Includes oily/tinned fish and white fish scores)	0.8 (0.3–1.3)	1.3 (0.8–1.5)	1.5 (1.3–2.0)	2.0 (1.5–2.0)	<0.001	Median: 1.5 Q1: 0.0–1.0 Q4: 1.8–2.0	Median: 1.3 Q1: 0.0–0.8 Q4: 1.8–2.0	<0.001
Unprocessed Meat Intake Score (Includes unprocessed red meat and poultry)	1.3 (1.0–1.7)	1.3 (1.0–1.7)	1.3 (1.3–1.7)	1.7 (1.3–1.7)	<0.001	Median: 1.3 Q1: 0.0–1.3 Q4: 1.7–2.0	Median: 1.3 Q1: 0.0–1.3 Q4: 1.7–2.0	<0.001
Other Protein Intake Score (Includes eggs, nuts, and beans/legumes)	1.5 (1.0–2.0)	2.0 (1.5–2.0)	2.0 (1.5–2.5)	2.5 (2.0–3.0)	<0.001	Median: 2.0 Q1: 0.0–1.5 Q4: 2.5–3.0	Median: 2.0 Q1: 0.0–1.5 Q4: 2.5–3.0	<0.001
Dairy Intake Score (Includes cow’s milk, yoghurt, and cheese)	1.3 (1.0–1.8)	1.8 (1.5–2.2)	2.0 (1.8–2.5)	2.5 (2.0–2.8)	<0.001	Median: 2.0 Q1: 0.0–1.5 Q4: 2.5–3.0	Median: 1.8 Q1: 0.0–1.3 Q4: 2.3–3.0	<0.001
Beverage Intake Score (Water as predominant beverage)	1.0 (0.5–1.0)	1.0 (0.5–1.0)	1.0 (1.0–1.0)	1.0 (1.0–1.0)	<0.001	Median: 1.0 Q1: 0.0–1.0 Q4: 1.0–1.0	Median: 1.0 Q1: 0.0–0.5 Q4: 1.0–1.0	<0.001
Olive Oil Score	0.0 (0.0–1.0)	1.0 (0.0–1.0)	1.0 (0.0–1.0)	1.0 (1.0–1.0)	<0.001	Median: 1.0 Q1: 0.0–0.0 Q4: 1.0–1.0	Median: 1.0 Q1: 0.0–0.0 Q4: 1.0–1.0	<0.001
Processed Snack Intake Score	0.0 (0.0–0.0)	0.0 (0.0–0.0)	0.0 (0.0–0.0)	0.0 (0.0–0.0)	<0.001	Median: 0.0 Q1: 0.0–0.0 Q4: 0.0–1.0	Median: 0.0 Q1: 0.0–0.0 Q4: 0.0–1.0	0.060
Processed Meat and ‘Junk food’ Score	0.0 (0.0–0.3)	0.0 (0.0–0.3)	0.0 (0.0–0.3)	0.0 (0.0–0.7)	<0.001	Median: 0.0 Q1: 0.0–0.0 Q4: 0.3–1.0	Median: 0.0 Q1: 0.0–0.0 Q4: 0.0–1.0	<0.001

Data presented as median and IQR. MDS: Mediterranean diet score. Data analysed via the following tests: ^a^ Kruskall–Wallis H test and ^b^ Mann–Whitney U test.

**Table 3:** ASPREE-MDS baseline characteristics by quartiles.

ASPREE-MDS	Q1 (3.3–9.8) (*n* = 3102)	Q2 (9.8–11.3) (*n* = 3096)	Q3 (11.3–12.6) (*n* = 3110)	Q4 (12.6–16.4) (*n* = 3086)	*p*
**Age (Median [IQR]), years**	77.3 (74.8–80.9)	77.0 (74.7–80.5)	76.7 (74.5–80.1)	76.3 (74.4–79.6)	<0.001 ^a^
**Female Sex, (n, %)**	1293 (41.7%)	1650 (53.2%)	1762 (56.7%)	2021 (65.8%)	<0.001 ^b^
**Caucasian Ethnicity, n (%)**	3066 (98.9%)	3063 (98.8%)	3072 (98.8%)	3041 (98.5%)	0.543 ^b^
**Education Completion**					<0.001 ^b^
≤11 years of education, (n, %)	1779 (57.4%)	1571 (50.7%)	1420 (45.7%)	1226 (39.7%)	
12 years of education, (n, %)	349 (11.3%)	329 (10.6%)	335 (10.8%)	327 (10.6%)	
≥13 years of education, (n, %)	971 (31.3%)	1199 (38.7%)	1355 (43.6%)	1533 (49.7%)	
**Lifestyle Factors**					
Currently Drinking Alcohol (n, %)	2123 (68.6%)	2294 (74.0%)	2350 (75.6%)	2362 (76.6%)	<0.001 ^b^
Currently Smoking (n, %)	130 (4.2%)	70 (2.3%)	62 (2.0%)	33 (1.1%)	<0.001 ^b^
**Living Situation**					0.057 ^b^
Home alone (n, %)	1071 (34.6%)	1018 (32.8%)	975 (31.4%)	1032 (33.4%)	
Home with spouse/friends/family (n, %)	2028 (65.4%)	2081 (67.2%)	2135 (68.6%)	2054 (66.6%)	
**BMI** (mean ± SD), kg/m^2^	27.9 ± 4.5	27.9 ± 4.7	27.5 ± 4.5	27.2 ± 4.5	0.085 ^c^ <0.001 ^b^
**BMI Categories** (n, %)					
BMI < 23 kg/m^2^	393 (12.7%)	379 (12.3%)	454 (14.6%)	508 (16.5%)	
BMI 23–28 kg/m^2^	1304 (42.2%)	1383 (44.7%)	1412 (45.5%)	1409 (45.7%)	
BMI 28–33 kg/m^2^	994 (32.1%)	930 (30.1%)	899 (28.9%)	853 (27.7%)	
BMI > 33 kg/m^2^	401 (13.0%)	401 (13.0%)	341 (11.0%)	314 (10.2%)	
**Abdominal circumference** (mean ± SD), cm	98.6 ± 12.6	97.3 ± 12.7	95.8 ± 12.6	94.0 ± 12.5	0.768 ^c^
Central adiposity (abnormal abdominal circumference (n, %)	1778 (57.4%)	1814 (58.5%)	1686 (54.2%)	1611 (52.2%)	<0.001 ^b^
**Laboratory Parameters**					
Glucose (mean ± SD), mg/dL	100.8 ± 19.7	99.9 ± 19.0	98.6 ± 17.0	98.0 ± 16.7	<0.001 ^c^
Total Cholesterol (mean ± SD), mg/dL	192.0 ± 37.8	197.1 ± 38.4	199.4 ± 38.8	202.0 ± 38.1	0.480 ^c^
HDL Cholesterol (mean ± SD), mg/dL	58.9 ± 17.4	60.5 ±17.6	62.7 ± 17.8	64.4 ± 18.1	0.112 ^c^
LDL Cholesterol (mean ± SD), mg/dL	108.6 ± 33.8	112.8 ± 34.0	113.7 ± 34.3	115.4 ± 34.1	0.867 ^c^
Triglycerides (mean ± SD), mg/dL	122.7 ± 58.3	120.0 ± 55.8	114.8 ± 56.2	110.9 ± 50.8	<0.001 ^c^
eGFR (mean ± SD), mL/min/1.73 m^2^	68.5 ± 14.9	70.1 ± 13.8	71.0 ± 13.9	72.0 ± 13.4	<0.001 ^c^
**Cardiometabolic Conditions**					
T2DM, n (%)	369 (11.9%)	343 (11.1%)	286 (9.2%)	281 (9.1%)	<0.001 ^b^
Hypertension, n (%)	2380 (76.8%)	2329 (75.2%)	2256 (72.5%)	2161 (70.0%)	<0.001 ^b^
Chronic Kidney Disease, n (%)	1041 (33.6%)	928 (29.9%)	860 (27.7%)	773 (25.0%)	<0.001 ^b^
Dyslipidaemia, n (%)	2496 (80.5%)	2553 (82.4%)	2606 (83.8%)	2568 (83.2%)	0.005
**Physical Function**					
Deficit-Accumulation Frailty Index					<0.001 ^b^
Not Frail	1439 (46.7%)	1513 (49.0%)	1611 (52.0%)	1728 (56.2%)	
Pre-Frail	1222 (39.7%)	1200 (38.8%)	1168 (37.7%)	1056 (34.4%)	
Frail	418 (13.6%)	376 (12.2%)	321 (10.4%)	290 (9.4%)	
Gait Speed (mean ± SD), m/s	1.1 ± 0.3	1.1 ± 0.3	1.0 ± 0.3	1.0 ± 0.3	<0.001 ^c^
Low Gait Speed, n (%)	266 (8.6%)	311 (10.0%)	352 (11.3%)	393 (12.7%)	<0.001 ^b^
Grip Strength (mean ± SD), kg	27.3 ± 9.7	26.3 ± 9.5	26.3 ± 9.5	25.2 ± 9.0	0.001 ^c^
Low Grip Strength, n (%)	717 (23.2%)	637 (20.6%)	567 (18.2%)	549 (17.8%)	<0.001 ^b^
**Neurocognitive Health**					
3MS Score (mean ± SD)	93.3 ± 5.2	94.2 ± 4.6	94.4 ± 4.7	95.0 ± 4.3	<0.001 ^c^
CESD score ≥ 8, n (%)	549 (18.4%)	511 (17.0%)	516 (17.1%)	443 (14.8%)	0.003 ^b^
**Dietary Scores**					
ASPREE-UPF (Median [IQR])	5.9 (4.8–7.1)	6 (4.9–7.4)	6.3 (5–7.4)	6.1 (5–7.3)	<0.001 ^a^

BMI: body mass index, T2DM: Type 2 diabetes mellitus, 3MS: Modified Mini Mental State Examination Score, CESD: Center for Epidemiological Studies Depression Scale, MDS: Mediterranean diet score, UPF: Ultra-processed food. Data analysed via ^a^ Kruskall–Wallis H Test, ^b^ Chi-squared test, ^c^ one-way ANOVA.

**Table 4:** ASPREE-UPF dietary components.

ASPREE-UPF Dietary Components	Q1 (0.3–4.9) (*n* = 3037)	Q2 (5.0–6.0) (*n* = 3009)	Q3 (6.1–7.3) (*n* = 3034)	Q4 (7.3–14.7) (*n* = 2882)	*p* ^a^	Female (Median: 5.8) (*n* = 6470)	Male (Median: 6.5) (*n* = 5492)	*p* ^b^
Sweetened/Processed Drinks (Includes malt, hot chocolate, cordial, soft drink, diet soft drink, and supplemental drinks)	0.0 (0.0–0.3)	0.0 (0.0–0.3)	0.3 (0.0–0.5)	0.5 (0.3–0.8)	<0.001	Median: 0.0 Q1: 0.0–0.0 Q4: 0.8–6.0	Median: 0.3 Q1: 0.0–0.0 Q4: 1.0–6.0	<0.001
Other Processed Drinks (Includes non-dairy milk and juice)	0.0 (0.0–0.3)	0.3 (0.0–0.5)	0.3 (0.0–0.5)	0.5 (0.3–0.8)	<0.001	Median: 0.3 Q1: 0.0–0.0 Q4: 0.8–2.0	Median: 0.3 Q1: 0.0–0.0 Q4: 0.8–2.0	<0.001
Breads (Includes white and brown breads)	0.8 (0.5–1.0)	1.0 (0.8–1.0)	1.0 (0.8–1.0)	1.0 (1.0–1.3)	<0.001	Median: 1.0 Q1: 0.0–0.5 Q4: 1.3–2.0	Median: 1.0 Q1: 0.0–0.8 Q4: 1.5–2.0	<0.001
Processed Meats and Related Foods (Includes burgers/pizza, pies, pre-packaged meals, sausages, and other processed meats)	0.5 (0.3–0.8)	0.8 (0.5–1.0)	1.0 (0.8–1.3)	1.4 (1.0–1.7)	<0.001	Median: 0.8 Q1: 0.0–0.3 Q4: 1.5–3.8	Median: 1.2 Q1: 0.0–0.5 Q4: 1.8–3.4	<0.001
Savoury Snacks (Includes crackers and chips)	0.3 (0.3–0.5)	0.5 (0.3–0.8)	0.8 (0.5–1.0)	0.8 (0.8–1.0)	<0.001	Median: 0.5 Q1: 0.0–0.0 Q4: 1.0–2.0	Median 0.8 Q1: 0.0–0.0 Q4: 1.3–2.0	<0.001
Sweetened Foods (Includes cakes, dark and milk chocolate, sweets/candy, cereal, and ice cream)	1.5 (1.0–1.8)	2.0 (1.8–2.5)	2.5 (2.25–3.0)	3.1 (2.8–3.5)	<0.001	Median: 2.3 Q1: 0.0–1.0 Q4: 3.3–5.3	Median: 2.5 Q1: 0.0–1.3 Q4: 3.5–5.5	<0.001
Processed Dairy (Includes yoghurt and cream cheese)	0.5 (0.3–1.0)	0.8 (0.5–1.0)	0.8 (0.5–1.3)	1.0 (0.5–1.3)	<0.001	Median: 0.8 Q1: 0.0–0.3 Q4: 1.3–2.0	Median 0.8 Q1: 0.0–0.0 Q4: 1.3–2.0	<0.001

Data presented as median and IQR. UPF: Ultra-processed food. Data analysed with ^a^ Kruskall–Wallis H test and ^b^ Mann–Whitney U test.

**Table 5:** ASPREE-UPF baseline characteristics by quartiles.

ASPREE-UPF	Q1 (0.3–4.9) (*n* = 3037)	Q2 (5.0–6.0) (*n* = 3009)	Q3 (6.1–7.3) (*n* = 3034)	Q4 (7.3–14.7) (*n* = 2882)	*p*
**Age (Median [IQR]), years**	76.5 (74.5–79.7)	76.6 (74.6–80.0)	76.9 (74.6–80.3)	77.0 (74.6–80.7)	<0.001 ^a^
**Female Sex, (n, %)**	1959 (64.5%)	1741 (57.9%)	1585 (52.2%)	1185 (41.1%)	<0.001 ^b^
**Caucasian Ethnicity, n (%)**	2979 (98.1%)	2974 (98.8%)	3003 (99.0%)	2862 (99.3%)	<0.001 ^b^
**Education Completion**					0.126 ^b^
≤11 years of education, (n, %)	1432 (47.2%)	1484 (49.3%)	1475 (48.6%)	1335 (46.3%)	
12 years of education, (n, %)	359 (11.8%)	305 (10.1%)	321 (10.6%)	312 (10.8%)	
≥13 years of education, (n, %)	1246 (41.0%)	1220 (40.5%)	1238 (40.8%)	1234 (42.8%)	
**Lifestyle Factors**					
Currently Drinking Alcohol (n, %)	2226 (73.3%)	2224 (73.9%)	2270 (74.8%)	2142 (74.3%)	0.566 ^b^
Currently Smoking (n, %)	106 (3.5%)	74 (2.5%)	57 (1.9%)	47 (1.6%)	<0.001 ^b^
**Living Situation**					<0.001 ^b^
Home alone (n, %)	1189 (39.2%)	1026 (34.1%)	930 (30.7%)	776 (26.9%)	
Home with spouse/friends/family (n, %)	1848 (60.8%)	1983 (65.9%)	2104 (69.3%)	2106 (73.1%)	
**BMI** (mean ± SD), kg/m^2^	27.5 ± 4.7	27.7 ± 4.5	27.7 ± 4.6	27.6 ± 4.3	0.006 ^c^ 0.003 ^b^
**BMI Categories** (n, %)					
BMI < 23 kg/m^2^	491 (16.2%)	403 (13.4%)	406 (13.4%)	368 (12.8%)	
BMI 23–28 kg/m^2^	1289 (42.6%)	1323 (44.1%)	1354 (44.6%)	1350 (46.9%)	
BMI 28–33 kg/m^2^	879 (29.0%)	927 (30.9%)	908 (29.9%)	836 (29.0%)	
BMI > 33 kg/m^2^	370 (12.2%)	349 (11.6%)	365 (12.0%)	327 (11.4%)	
**Abdominal circumference** (mean ± SD), cm	94.9 ± 12.9	96.3 ± 12.7	96.9 ± 12.8	97.9 ± 12.2	0.019 ^c^
Central adiposity (abnormal abdominal circumference (n, %)	1703 (56.1%)	1737 (57.7%)	1672 (55.1%)	1545 (53.6%)	0.013 ^b^
**Laboratory Parameters**					
Glucose (mean ± SD), mg/dL	98.5 ± 17.0	99.6 ± 19.4	99.5 ± 17.8	99.6 ± 17.7	<0.001 ^c^
Total Cholesterol (mean ± SD), mg/dL	200.5 ± 39.4	198.8 ± 38.8	196.5 ± 37.9	194.5 ± 37.4	0.019 ^c^
HDL Cholesterol (mean ± SD), mg/dL	64.3 ± 18.1	62.6 ± 17.8	60.8 ± 17.7	58.7 ± 17.2	0.073 ^c^
LDL Cholesterol (mean ± SD), mg/dL	113.3 ± 34.6	112.8 ± 34.7	111.8 ± 33.7	112.4 ± 33.4	0.081 ^c^
Triglycerides (mean ± SD), mg/dL	114.8 ± 53.8	116.8 ± 54.5	119.2 ± 58.1	117.7 ± 55.3	<0.001 ^c^
eGFR (mean ± SD), mL/min/1.73 m^2^	71.1 ± 14.0	70.9 ± 13.8	70.1 ± 14.1	69.7 ± 14.2	0.556 ^c^
**Cardiometabolic Conditions**					
T2DM, n (%)	298 (9.8%)	291 (9.7%)	341 (11.2%)	289 (10.0%)	0.165 ^b^
Hypertension, n (%)	2253 (74.2%)	2226 (74.0%)	2224 (73.3%)	2101 (72.9%)	0.655 ^b^
Chronic Kidney Disease, n (%)	860 (28.3%)	846 (28.1%)	884 (29.1%)	866 (30.0%)	0.343 ^b^
Dyslipidaemia, n (%)	2555 (84.1%)	2515 (83.6%)	2489 (82.0%)	2314 (80.3%)	<0.001 ^b^
**Physical Function**					
Deficit-Accumulation Frailty Index					0.183 ^b^
Not Frail	1576 (52.1%)	1589 (53.0%)	1508 (49.8%)	1452 (50.6%)	
Pre-Frail	1131 (37.4%)	1097 (36.6%)	1162 (38.4%)	1087 (37.9%)	
Frail	319 (10.5%)	312 (10.4%)	356 (11.8%)	331 (11.5%)	
Gait Speed (mean ± SD), m/s	1.1 ± 0.3	1.0 ± 0.3	1.1 ± 0.3	1.1 ± 0.3	0.007 ^c^
Low Gait Speed (n, %)	335 (11.0%)	355 (11.8%)	310 (10.2%)	300 (10.4%)	0.193 ^b^
Grip Strength (mean ± SD), kg	25.1 ± 8.9	26.0 ± 9.3	26.6 ± 9.5	28.0 ± 9.9	<0.001 ^c^
Low Grip Strength (n, %)	544 (17.9%)	567 (18.8%)	612 (20.2%)	606 (21.0%)	0.013 ^b^
**Neurocognitive Health**					
3MS Score (mean ± SD)	94.4 ± 4.7	94.7 ± 4.5	94.3 ± 4.8	94.1 ± 4.8	0.001 ^c^
CESD score ≥ 8, n (%)	437 (14.9%)	457 (15.7%)	498 (17.0%)	530 (19.0%)	<0.001 ^b^
**Dietary Scores**					
ASPREE-MDS (Median [IQR])	11.1 (9.6–12.5)	11.3 (9.9–12.6)	11.4 (9.9–12.6)	11.4 (10.1–12.6)	<0.001 ^a^

BMI: body mass index, T2DM: Type 2 diabetes mellitus, 3MS: Modified Mini Mental State Examination Score, CESD: Center for Epidemiological Studies Depression Scale, MDS: Mediterranean diet score, UPF: Ultra-processed food. Data analysed via ^a^ Kruskall–Wallis H Test, ^b^ Chi-squared test, ^c^ one-way ANOVA.

**Table 6:** Cardiometabolic diseases, frailty markers, and dietary patterns.

	ASPREE-MDS		ASPREE-UPF	
	Univariate	Multivariate	Univariate	Multivariate
T2DM *	OR 0.94 (95% CI 0.91–0.96)	aOR 0.99 (95% CI 0.96–1.02)	OR 1.01 (95% CI 0.98–1.04)	aOR 0.98 (95% CI 0.95–1.02)
Hypertension *	OR 0.93 (95% CI 0.91–0.95)	aOR 0.96 (95% CI 0.94–0.98)	OR 0.98 (95% CI 0.96–1.01)	aOR 0.97 (95% CI 0.94–0.99)
CKD *	OR 0.91 (95% CI 0.90–0.94)	aOR 0.94 (95% CI 0.92–0.96)	OR 1.02 (95% CI 1.00–1.05)	aOR 1.01 (95% CI 0.98–1.03)
Dyslipidaemia *	OR 1.03 (95% CI 1.01–1.06)	aOR 0.99 (95% CI 0.97–1.02)	OR 0.94 (95% CI 0.91–0.96)	aOR 0.98 (95% CI 0.95–1.01)
Deficit-Accumulation Frailty Index †				
Not Frail	*Reference*	*Reference*	*Reference*	*Reference*
Pre-Frail	RR 0.93 (95% CI 0.91–0.95)	aRR 0.93 (95% CI 0.91–0.95)	RR 1.01 (95% CI 0.99–1.03)	aRR 1.04 (95% CI 1.01–1.06)
Frail	RR 0.89 (95% CI 0.86–0.91)	aRR 0.88 (95% CI 0.86–0.91)	RR 1.04 (95% CI 1.00–1.07)	aRR 1.10 (95% CI 1.06–1.14)
Deficit-Accumulation Frailty Index ^§^				
Not Frail		*Reference*		*Reference*
Pre-Frail		aRR 0.93 (95% CI 0.91–0.95)		aRR 1.05 (95% CI 1.02–1.07)
Frail		aRR 0.87 (95% CI 0.84–0.90)		aRR 1.11 (95% CI 1.07–1.16)

Dietary scores as continuous variables. T2DM: Type 2 diabetes mellitus, CKD: Chronic kidney disease, MDS: Mediterranean diet score, UPF: Ultra-processed food. Multivariate analysis adjusted for the following: age at time of FFQ, sex, BMI category (<23, 23–28, 28–33, >33), educational attainment, living status (alone vs. with others), current smoking status, and current drinking status. * Calculated using logistic regression. † Calculated using multinomial logistic regression using the multivariate adjustment above. ^§^ Calculated using multinomial logistic regression using the multivariate adjustment above as well as the other score (ASPREE-UPF included in the ASPREE-MDS analysis and vice versa).

## Data Availability

The original contributions presented in this study are included in the article/Supplementary Material, further inquiries can be directed to the corresponding author/s.

## Supplementary Materials

The following supporting information can be downloaded at: https://www.mdpi.com/article/10.3390/nu16172978/s1, Table S1: Food frequency questionnaire used in ASPREE longitudinal study of older persons (ALSOP); Table S2: ASPREE-UPF components and scoring; Table S3: ASPREE-MDS components and scoring.
