# Supplementary material for: Mediterranean Diet and Ultra-Processed Food Intake in Older Australian Adults—Associations with Frailty and Cardiometabolic Conditions
Source: Nutrients. 2024 Sep 3;16(17):2978. doi: 10.3390/nu16172978 (PMC11397489; doi:10.3390/nu16172978)
Supplement: Supplementary file 1 [file nutrients-16-02978-s001.zip › nutrients-3174833-supplementary.pdf]

Supplementary Table S1. Food frequency questionnaire used in ASPREE longitudinal study of older persons (ALSOP)

**Food Item**	**Never/ rarely**	**Once or twice a month**	**Once or twice a week**	**Often or 3-6 times per week**	**Every day or several times a day**
Cheese					
Yoghurt					
Cream, cream cheese or similar					
Ice cream, frozen yoghurt or other dairy desserts					
White bread					
Brown/multigrain bread					
Breakfast cereal/ oats					
Rice					
Pasta / noodles					
Crackers / savoury biscuits					
Fresh fruit					
Cooked, tinned or dried fruit					
Raw/salad vegetables					
Cooked green vegetables					
Cooked orange / yellow / red vegetables e.g. carrots, beets					
Potatoes					

Beans and Legumes<br>e.g. peas, lentils					
Red meat (not corned, pickled<br>or processed)					
Chicken, turkey, other poultry					
Sausages					
Processed meats e.g. bacon,<br>ham, corned beef or salami					
Eggs					
Deep fried/battered fish					
Salmon, mackerel or other oily fish<br>not including deep fried fish					
White fish,<br>not including deep fried fish					
Tinned fish e.g. canned salmon,<br>tuna, sardines					
Other seafood e.g. prawns or<br>shellfish					
Nuts					
Potato chips or similar					
Sweet biscuits/ cakes					
Dark Chocolate					
Milk Chocolate					
Lollies or other sweets					

Hamburgers, pizza<br>or 'fast' food					
Meat pies, sausage rolls etc					
Drinks	**Never/ rarely**	**Once per week or less**	**Several times a week**	**1-2 times a day**	**3 or more times / day**
Full-cream milk					
Low-fat or skim milk					
Soy or other non-dairy milk					
Water					
Coffee					
Tea					
Malt drinks e.g. Milo or Horlicks					
Hot chocolate					
Fruit juice					
Cordial					
Soft Drink e.g. regular Coke					
Diet Soft drink e.g. diet Coke					
Supplement drink<br>e.g. Ensure or Sustagen					
Herbal tea					

Which was the most common cooking oil used in your household? (Select one only)	**Yes**	**No**
Olive oil		
Butter		
Other vegetable oil		
Don’t know		
Did you regularly add salt to your cooking?		
Did you regularly add salt to your food at the table?		

* Response recorded for question: Please think about your diet over the last 12 months. How often did you eat the following foods?

Supplementary Table S2. ASPREE-UPF components and scoring.

Over the past year, how often did you have a *cup* of the following drinks?	Never/Rarely	Once per week or less	Several times a week	1-2 times per day	3 or more times per day
Malt drinks (e.g., Milo or Horlicks)	0	0.25	0.5	0.75	1
Alternative Milks	0	0.25	0.5	0.75	1
Hot Chocolate	0	0.25	0.5	0.75	1
Cordial	0	0.25	0.5	0.75	1
Juice	0	0.25	0.5	0.75	1
Soft Drink	0	0.25	0.5	0.75	1
Diet Soft Drink	0	0.25	0.5	0.75	1
Supplemental Drinks (e.g., Sustagen)	0	0.25	0.5	0.75	1

	Never/Rarely	1-3 times per month	1 day per week	2-3 days per week	4-6 days per week	Every day
Over the past year, how often do you eat pre-prepared	0	0.2	0.4	0.6	0.8	1

meals? E.g., those bought frozen or home-delivered that you might reheat at home						

Please think about your diet over the last **12 months**. How often did you eat the following foods?	Never/Rarely	Once or twice a month	Once or twice a week	Often 3-6 times per week	Every day or several times per day
Sausages	0	0.25	0.5	0.75	1
Processed Meats (e.g., bacon, ham, corned beef or salami)	0	0.25	0.5	0.75	1
Potato chips (or similar)	0	0.25	0.5	0.75	1
Sweet biscuits / cake	0	0.25	0.5	0.75	1
Dark Chocolate	0	0.25	0.5	0.75	1
Milk Chocolate	0	0.25	0.5	0.75	1
Lollies or other sweets	0	0.25	0.5	0.75	1
Burgers, Pizza, or ‘fast food’	0	0.25	0.5	0.75	1

Meat Pies, Sausage Rolls, etc.	0	0.25	0.5	0.75	1
Ice-cream, frozen yoghurt, or other dairy desserts	0	0.25	0.5	0.75	1
White bread	0	0.25	0.5	0.75	1
Brown/multigrain bread	0	0.25	0.5	0.75	1
Cereal / Oats	0	0.25	0.5	0.75	1
Crackers / savoury biscuits	0	0.25	0.5	0.75	1
Yoghurt	0	0.25	0.5	0.75	1
Cream, Cream Cheese, or similar	0	0.25	0.5	0.75	1

Items Not Included: Whole milk, skim/light milk, water, coffee, tea, fruit juice, herbal tea, cooking oil (olive oil vs butter vs other vegetable oil vs do not know), addition of salt to cooking, addition of salt to table, missed meal frequency, unprocessed red meat, chicken/poultry, eggs, fried/battered fish, oily fish, tinned fish, white fish, prawns/shellfish, nuts, cheese, rice, pasta/noodles, fresh fruit, tinned/dried/cooked fruit, raw/salad vegetables, cooked green vegetables, cooked orange/yellow/red vegetables, potatoes, legumes/beans.

The total **ASPREE-UPF** score is the sum of each of the scores attributable to each of these UPFs.

Supplementary Table S3. ASPREE-MDS components and scoring.

**Food Category**	**Never**	**1-2/Month**	**1-2/Week**	**3-6/Week**	**Daily or more**	Scoring System
Green Vegetables						Sum all to a maximum of 1.5 points, then divide by 1.5
Raw / Salad Vegetables	0	0	0.33	0.67	1	
Cooked Green Vegetables	0	0	0.33	0.67	1	**Maximum of 1 point**
Other Vegetables						Sum all to a maximum of 1.5 points, then divide by 1.5
Cooked Red/Orange/Yellow Veg	0	0	0.33	0.67	1	
Potatoes	0	0	0.33	0.67	1	**Maximum of 1 point**
Grains						Sum all to a maximum of 3 points, then divide by 3
Brown Bread*	0	0	0.33	0.67	1	
Cereal and Oats	0	0	0.33	0.67	1	
Rice	0	0	0.33	0.67	1	**Maximum of 1 point**
Pasta / Noodles	0	0	0.33	0.67	1	
Fruit						Sum All
Fresh Fruit	0	0	0.33	0.67	1	**Maximum of 1**
Tinned/Dried Fruit	0	0	0.33	0.5	0.5	
Chicken/Poultry	0	0.33	1	0.67	0.33	**Maximum of 1**
Red Meat (unprocessed)	1	1	0.67	0.33	0	**Maximum of 1**

Nuts	0	0	0.5	1	1	**Maximum of 1**
Beans/Legumes	0	0	0.5	1	1	**Maximum of 1**
Oily Seafood**<br>Oily Fish<br>Tinned Fish<br>Other Seafood<br>White Fish<br>Other Seafood	 0<br>0<br><br>0<br>0	 0.5<br>0.5<br><br>0.25<br>0.25	 1<br>1<br><br>0.5<br>0.5	 1.5<br>1.5<br><br>0.5<br>0.5	 1.5<br>1.5<br><br>0.5<br>0.5	Oily Fish Capped at 1.5<br><br>Other Fish Capped at 0.5<br><br>Total Seafood Points = Oily + Other, **Capped at 2 total**
Eggs	0	0.5	1	1	0.5	**Maximum of 1**
Cheese***	0	0.5	1	1	0.5	**Maximum of 1**
Yoghurt	0	0.5	1	1	1	**Maximum of 1**
Predominant Cooking Fat<br>Olive Oil<br>(Other)						1 Point if Olive Oil, else 0<br><br>**Maximum of 1**
**Drinks Category**	**Never/Rarely**	**Once per week or less**	**Several times per week**	**1-2 times per day**	**3 or more times per day**	
Milk****<br>Full Cream<br>Skim/Reduced Fat	 0<br>0	 0.33<br>0.33	 0.75<br>0.75	 1<br>1	 0.5<br>0.5	1. If one is never, then the other score stands<br>2. If both are 1-2 times per day, 0.75

						3. If one is 3 or more times per day and the other is more than never, 0.5<br>4. If both are several times per week, 1<br>5. All other cases, average the two scores<br><br>**Maximum of 1**
Beverage Choices<br>Water<br>Cordial<br>Juice<br>Soft Drink<br>Diet Soft Drink						If water is drunk with the most frequency, 1 point.<br>If it's equal highest, 0.5 points.<br>Else, 0 points.<br><br>**Maximum of 1**
Snack and Processed Foods Calculated Differently						
**Negative Food Category**	**Never**	**1-2/Month**	**1-2/Week**	**3-6/Week**	**Daily or more**	
Snack Foods						Sum all values, maximum value of 1
Chips	0	0.33	0.67	1	1	
Cakes/Pastries	0	0.33	0.67	1	1	

Dark Chocolate	0	0.33	0.67	1	1	Then, ASPREE-MDS is calculated as 1 – Summed Score
Milk Chocolate	0	0.33	0.67	1	1	
Sweets/Lollies	0	0.33	0.67	1	1	
Savoury Crackers	0	0.33	0.67	1	1	**Maximum of 1**
Takeaway & Processed Foods						Sum all values, maximum value of 1
Burgers/Pizza	0	0.33	0.67	1	1	
Pies	0	0.33	0.67	1	1	
Sausages	0	0.33	0.67	1	1	Then, ASPREE-MDS is calculated as 1 – Summed Score
Other Processed Meats	0	0.33	0.67	1	1	
						**Maximum of 1**

Total Possible = 18 Points

* Only brown bread—white bread not included;

** Excludes deep-fried/battered fish;

*** Excluding cream cheese, etc.;

**** Excluding soy milk or other alternative milks.
